# Treatment Strategies and Outcomes of Pediatric Esthesioneuroblastoma: A Systematic Review

**DOI:** 10.3389/fonc.2020.01247

**Published:** 2020-07-24

**Authors:** Chetan Safi, Daniel Spielman, Marc Otten, Jeffrey N. Bruce, Neil Feldstein, Jonathan B. Overdevest, David A. Gudis

**Affiliations:** ^1^Department of Otolaryngology – Head and Neck Surgery, New York-Presbyterian Hospital – Columbia University Irving Medical Center, New York, NY, United States; ^2^Department of Neurologic Surgery, New York-Presbyterian Hospital – Columbia University Irving Medical Center, New York, NY, United States

**Keywords:** esthesioneuroblastoma, olfactory neuroblastoma, pediatric skull base surgery, endoscopic skull base surgery, skull base tumor, pediatric neuroendoscopic surgery, head and neck cancer, skull base cancer

## Abstract

**Introduction:** Esthesioneuroblastoma, also known as olfactory neuroblastoma, is a small round blue cell tumor of nasal neuroepithelium first described in 1924. Though this tumor is especially rare in the pediatric population with an incidence of <0.1 per 100,000, it is the most common pediatric nasal cavity neoplasm. The purpose of this systematic review is to examine the treatment modalities utilized for pediatric esthesioneuroblastoma and overall survival.

**Methods:** A systematic review was performed according to the Preferred Reporting Items for Systematic Reviews and Meta-Analysis (PRISMA) guidelines. Pubmed, EMBASE, and Ovid MEDLINE databases were queried for studies pertinent to treatment modalities for pediatric esthesioneuroblatoma and survival outcomes.

**Results:** Two hundred and seventy-sixth articles were identified, with seven meeting inclusion criteria. Ninety-four patients with an age range of 0.9–21 years old with esthesioneuroblastoma were included. Nearly 90% of patients were of stage Kadish B or C at time of presentation, while 20% presented with cervical lymphadenopathy. Only about 10% of patients underwent single modality therapy. Overall, 5-year survival ranged from 44 to 91% with a median follow-up of 3–13 years.

**Conclusion:** Children with esthesioneuroblastoma usually present at an advanced stage and undergo multi-modality therapy at a higher rate than adult patients. There is a wide range of documented overall survival though this lack of precision could be due to a paucity of patients.

## Introduction

Esthesioneuroblastoma, also known as olfactory neuroblastoma, is a small round blue cell tumor of nasal neuroepithelium first described in 1924 (1). This tumor comprises about 28% of pediatric nasal cavity cancers and is the most common nasal cavity neoplasm in children (2). Presenting symptoms usually include nasal obstruction, facial pain, epistaxis, and visual and intracranial complications based on extent of tumor spread (3, 4). Computed tomography (CT) and magnetic resonance imaging (MRI) play a complimentary role in diagnosis, as CT provides information on osseous erosion while MRI provides insight into soft tissue spread (5). Adult patients are usually treated with surgical resection followed by postoperative radiation therapy (6). However, due to the rarity of this diagnosis in children, there is limited literature analyzing treatment algorithms. Thus, the purpose of this study is to examine the treatment modalities used for pediatric esthesioneuroblastoma and the overall survival of these patients.

## Methods

A comprehensive review of the English language-literature was performed from the PubMed, EMBASE, and Ovid MEDLINE databases through the OVID portal. The search was conducted using the phrase “pediatric esthesioneuroblastoma.” Inclusion criteria were defined using the Population, Intervention, Control, Outcome, and Study Design (PICOS; Table 1) approach (7). Studies included in the review were those with pediatric patients with a diagnosis of esthesioneuroblastoma and with documented survival data after undergoing treatment. Case series with fewer than five patients were excluded. A systematic search of the literature was performed using the Preferred Reporting Items for Systematic Reviews and Meta-analyses (PRISMA) literature selection process (8).

**Table 1:** Population, Intervention, Control, Outcome, Study Design (PICOS) Inclusion Criteria.

**Population**	**Inclusion criteria**	**Exclusion criteria**
	**Pediatric**	**Adults**
Intervention	1. Treatment of esthesioneuroblastoma including surgical excision, chemotherapy, and/or radiation therapy	1. No mention of treatment modality
Comparator	1. Evaluate the most commonly used treatment modalities for pediatric esthesioneuroblastoma	
Outcome	1. Disease free and overall survival for pediatric esthesioneuroblastoma	
Study design	Case series, retrospective, prospective	Case series with <5 patients, case reports

Two reviewers (C.S. and D.S.) independently examined all articles in a standardized manner to determine study eligibility and then compared highlighted articles. All duplicate records were removed. The abstract of every citation was screened for relevance to pediatric esthesioneuroblastoma. Case reports and irrelevant articles were discarded. Full text articles were then assessed for eligibility. Clinical studies with fewer than five patients and those without discussion of either treatment modality or overall survival were excluded. Furthermore, only manuscripts evaluating pediatric esthesioneuroblastoma exclusively were included to prevent ambiguity of data from series that included both pediatric and adult patients. The remaining articles meeting all inclusion and exclusion criteria were included for qualitative and quantitative analysis.

Data collected from each study included authors, year of publication, study design, patient demographics, patient population, number of patients, and treatment modalities used. Outcome measures examined included overall survival. A meta-analysis could not be performed due to the heterogeneity in reporting of treatment modalities and outcome measures.

## Results

**Figure 1:**
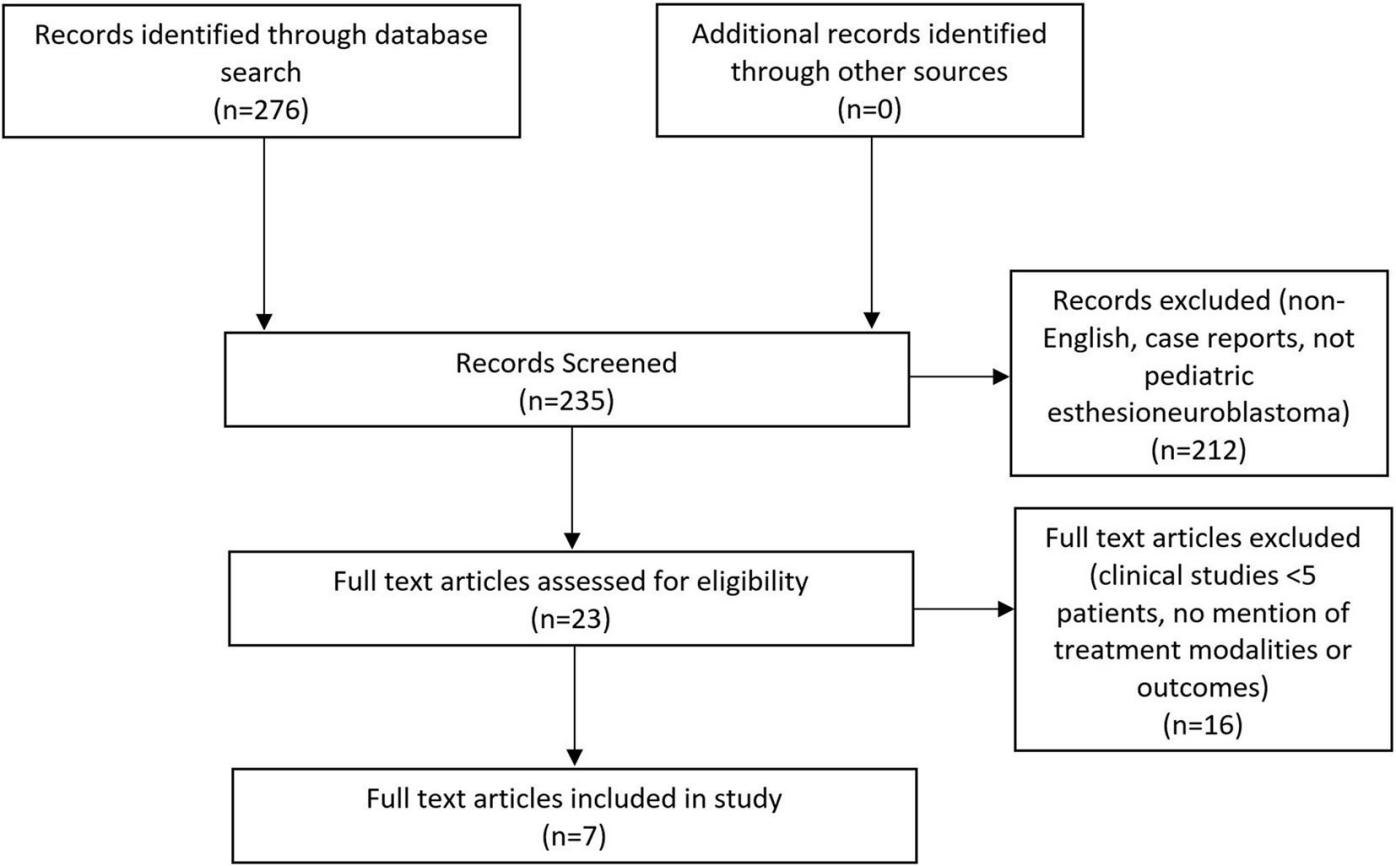
Preferred Reporting Items for Systematic Reviews and Meta-Analyses (PRISMA) flow diagram highlighting the literature selection process.

The initial database query identified 276 articles (Figure 1). After the duplicates were removed, the abstracts of the remaining 235 citations were screened for articles related to pediatric esthesioneuroblastoma that were not case reports. The remaining 23 articles from this initial screen underwent a full-text assessment for eligibility. Manuscripts that did not specify treatment modalities or lacked data on survival outcomes were excluded.

A total of seven articles met final inclusion criteria. A summary of these articles is found in Table 2. A total of 94 pediatric patients with an age range of 0.9–21 years and a male to female ratio of 43–57% were included in this study. All studies were retrospective case series and included patients treated between 1980 and 2014. There was a lack of uniformity amongst the articles in reporting how patients were diagnosed with esthesioneuroblastoma. Some simply described that the diagnosis was confirmed histologically while others were more detailed and explained that biopsy showed sheets and nests of round blue cells with scant cytoplasm. Some authors even described that immunohistochemistry of presumed masses stained positive for chromogranin, synaptophysin, and neuron-specific enolase. Studies did not uniformly comment on Hyam's histological grading. CT and MRI were used to determine the extent of spread of the mass and provide Kadish staging. 2.1% (2/94) of patients were Kadish A, 28.7% (27/94) were Kadish B, 60.6% (57/94) were Kadish C, and 8.5% (8/94) were Kadish D as seen in Table 3. Cervical lymph node metastases were found in 20.2% (19/94) of patients.

**Table 2:** Pediatric esthesioneuroblastoma treatment modalities and survival outcomes.

**Author, year**	**Timeline**	**Number of patients**	**Median age, yr (range)**	**M:F**	**Kadish stage**	**% with cervical LN metastases**	**Treatment**	**Median follow-Up, yr (range)**	**Survival**
Bisogno et al. 2012 (9)	1980–2008	9	9.9 (0.9–18)	6:3	B−3	22.2% (2/9)	Chemo/RT−3 Chemo/Surg−1 Triple Mod−5	13.4 (9.2–22.9)	PFS−77.8% (33.6–93.9%) OS−88.9% (43.3–98.4%)
Dumont et al. 2020 (10)	1990–2005	18	12.2 (0.9–18)	10:8	A−1 B−3 C−10 D−4	10.5% (2/19)	Surg/RT−4 Chemo−4 Chemo/RT−2 Chemo/Surg−3 Triple Mod−5	7.6 (3.8–17.9)	PFS = OS−44.4% (± 11.7%)
Eich et al. 2005 (3)	1979–2001	19	14 (5–20)	9:10	B−4 C−15	11.1% (2/18)	Surg−4 Surg/RT−1 Chemo/RT−2 Triple Mod−12	3.1 (0.25–23)	PFS−55% ± 13% OS−73% ± 12%
Kababri et al. 2014 (4)	1982–2002	11	14 (0.8–18)	3:8	B−5 C−6	9.1% (1/11)	Surg/RT−1 Chemo/RT−1 Chemo/Surg−1 Triple Mod−8	8.8 (3.8–16.4)	PFS = OS−91% (62–98%)
Kumar et al. 2002 (11)	1989–2000	5	13 (5–16)	4:1	A−1 B−1 C−3	20% (1/5)	Chemo/RT−2 Chemo−1 Chemo/Surg−1 Triple Mod−1	N/A	3/5 dead at 18 months after diagnosis
Lucas et al. 2015 (12)	2000–2013	8	10 (4–21)	2:6	B−3 C−1 D−4	37.5% (3/8)	Surg/RT−2 Chemo/RT−2 Triple Mod−4	4.6 (4–21)	OS−87.5%
Venkatramani et al. 2016 (13)	1990–2014	24	12 (0.6–20)	6:18	B−8 C−16	33.3% (8/24)	Surg−1 Surg/RT−10 Chemo/RT−1 Triple Mod−12	3.8 (0.5–21.9)	PFS−73.7% (50.5–87.3%) OS−72.8% (46–87.9%)

*M, Male; F, Female; LN, lymph node; Chemo, chemotherapy; RT, radiation therapy; Surg, surgery; Triple Mod, Triple Modality Therapy; PFS, Progression Free Survival; OS, Overall Survival*.

**Table 3:** Kadish stage upon diagnosis.

**Kadish Stage**	**# of patients (%)**
A	2/94 (2.1%)
B	27/94 (28.7%)
C	57/94 (60.6%)
D	8/94 (8.5%)

*Kadish Staging: A – tumor confined to nasal cavity; B – involvement of one or more paranasal sinuses; C – extension beyond the paranasal sinuses involving cribiform plate, skull base, or orbit; D – regional lymph node or distant metastasis*.

Each study was evaluated for the therapeutic modalities used to treat pediatric esthesioneuroblastoma including neoadjuvant and adjuvant chemotherapy (CT), radiation therapy (RT), and surgical resection, as seen in Table 4. For chemotherapy, authors described most commonly using a combination of agents including but not limited to vincristine, doxorubicin, cyclophosphamide, cisplatin, etoposide, and isofosfamide. These therapies were adapted from the chemotherapy protocols set in place at each institution and/or country for pathologies such as rhabdomyosarcoma, neuroblastoma, and Ewing's sarcoma. While authors were not specific about exact protocol used, one did mention that treatment would vary from five to 15 cycles. Chemotherapy was utilized in 75.5% of patients (71/94). Overall, radiation therapy was utilized in 83.0% of cases (78/94) and varied between proton therapy as well as more traditional photon radiotherapy. Most articles described using a median radiation dose of 50–60 Gray (Gy) with ~2 Gy per fraction for a median number of fractions ranging from 25 to 32. Only one study described using proton therapy while another specifically stated that linear accelerators were used to form anterior and wedged lateral radiation fields for the treatment field. Surgical resection was performed in 80.9% of cases (76/94) and involved endoscopic endonasal resection or craniofacial resection with or without craniotomy, as well as cervical lymphadenectomy when indicated.

**Table 4:** Treatment modalities.

**Treatment Modality**	**# of patients (%) *N* = 94 patients**
Surgery	5 (5.3%)
Chemo	5 (5.3%)
Chemo + RT	13 (13.8%)
Surgery + RT	18 (19.1%)
Surgery + Chemo	6 (6.4%)
Triple Modality Therapy	47 (50%)

*Chemo, chemotherapy; RT, radiation therapy*.

Single modality therapy was utilized in 10.6% of patients (5 surgery, 5 CT). Dual modality therapy was used in 39.4% of patients (18 surgery and RT, 13 CT and RT, 6 CT and surgery). Triple modality therapy was used in 50% (47/94) of patients. Both the progression free survival and overall survival ranged from 44.4 to 91% with median follow-up of 6.1 years. Five of the seven included studies have an overall survival >70% indicating an overall positive prognosis. Unfortunately, due to the heterogeneity of the reported data as well as varying treatment modalities utilized, a meta-analysis could not be performed.

## Discussion

Pediatric esthesioneuroblastoma is a very uncommon pathologic diagnosis. As a result, no single center or provider has significant treatment experience. Our aim in this systematic review was to synthesize the available literature to determine commonly used treatment modalities as well as overall survival.

Regarding patient presentation, only 2.1% of pediatric patients presented with a Kadish stage A tumor limited to the nasal cavity. In contrast, a study looking at over 800 patients from the National Cancer Database found that almost 22% of adults were diagnosed with a Kadish stage A tumor, possibly indicating that children present with a more aggressive phenotype of the disease (9). This is further supported by the fact that adult patients were found to have regional metastases to the cervical lymph nodes in 7.3% of patients while children and adolescents were found to have a 20.2% regional metastasis rate in our study. These data reinforce the concept that esthesioneuroblastoma in the pediatric population is a more aggressive tumor compared to adult disease. This finding emphasizes the importance of a unique treatment paradigm in managing pediatric esthesioneuroblastoma.

Treatment strategies differ significantly between pediatric and adult patients. Adult patients usually undergo surgical resection, radiation therapy, or a combination of both (3, 4, 12, 14). However, our study found that 50% of patients underwent triple modality therapy with surgical resection, CT, and RT, while only about 10% of patients underwent single modality therapy. Surprisingly, nearly half of adult patients in a large series underwent single modality therapy while extensive surgical excision, CT, and RT was reserved for <15% of patients (14). Furthermore, in another study involving 22 patients with esthesioneuroblastoma with a median age of 45, all patients underwent craniofacial resection as well as radiotherapy for treatment. Thirty-six percentage of patients underwent combination proton and photon radiotherapy while the rest underwent proton therapy. In this group, only 22.7% of patients were treated with chemotherapy such as etoposide, cisplatin, and carboplatin. These data further suggest the treatment dichotomy between adults and pediatric patients with esthesioneuroblastoma (15). Apart from a higher stage tumor requiring more aggressive therapy, these discrepancies in treatment modalities could also be related to the consideration of post-treatment sequelae for children. For example, young children have small nasal cavities making oncologic surgical resection challenging with vital structures such as the orbit and brain in such close proximity (13). Furthermore, radiation therapy of the head and neck in young children requires special consideration due to the potential for endocrine dysfunction and an increased risk of secondary malignancy later in life (13).

Regarding survival outcomes, we found an overall 5-year survival ranging from 44 to 91% with several years of follow-up in most studies. This finding is similar to several studies involving adult patients that demonstrate an overall 5-year survival ranging from about 60 to 95% (14–16). This wide range in overall survival could be explained by the relatively small number of patients involved in each study with a variety of treatment strategies, making the task of identifying an optimal treatment strategy even more challenging. A major limitation in this study is that all of the included publications were small retrospective case series. Each author used individualized treatment algorithms and varied outcome measures that prevent qualitative analysis. Furthermore, not every study commented on survival with respect to unique treatment modalities, so definitive conclusions about which treatment strategies were more beneficial could not be made. Due to the rarity of pediatric esthesioneuroblastoma, continuing to gather high volume data from several different institutions will be key in determining the optimal treatment strategy with the best outcome.

## Conclusion

Esthesioneuroblastoma is a rare small round blue cell tumor found in the nasal cavity and paranasal sinuses. Children appear to present with a more locally and regionally advanced tumor when compared to adults, likely predisposing providers to use more multimodality therapy. Moreover, it is possible that due to the long term sequelae of radiation therapy and extensive surgical resection, triple modality therapy is favored to provide a more balanced approach in treating the cancer and limiting complications. The overall 5-year survival in pediatric patients is varied and future studies are needed in order to determine the ideal treatment regimen that will limit lifelong morbidity in this young patient population.

## Data Availability Statement

The raw data supporting the conclusions of this article will be made available by the authors, without undue reservation.

## Author Contributions

All authors contributed to the concept development, data analysis, and manuscript composition and editing.

## Conflict of Interest

The authors declare that the research was conducted in the absence of any commercial or financial relationships that could be construed as a potential conflict of interest.
